# Perceptions of Temporal Selves: Continuity, Psychological Outcomes, and the Significance of a Disadvantaged Background

**DOI:** 10.3390/bs14100858

**Published:** 2024-09-24

**Authors:** Samantha L. McMichael, Kevin D. Redifer, Virginia S. Y. Kwan

**Affiliations:** Department of Psychology, Arizona State University, Tempe, AZ 85287, USA

**Keywords:** self-continuity, temporal self-perceptions, socioeconomic status, mental health, psychological well-being

## Abstract

Psychologists and philosophers have argued that a consistent self-concept is essential for mental health and well-being. Differences in individuals’ backgrounds—specifically, their financial resources—may be instrumental to understanding these relationships. This research: (1) tested the continuity of self-perceptions, (2) investigated if perceptions of the past and future self predicted depression and well-being, and (3) explored whether continuity and its relationship to psychological outcomes varied across SES. The findings suggest: (1) moderate continuity; (2) perceptions of past and future were unique predictors of psychological outcomes; and (3) significant differences in continuity and how it predicted meaning-in-life by SES. The results extend the understanding of continuity and its significance for psychological outcomes while establishing the importance of individuals’ backgrounds in these relationships.

## 1. Introduction

Traditionally, psychologists and Western philosophers have argued that individuals have a consistent, core self that is relatively stable over time [1,2]. A lack of continuity, or a perceived “sameness”, of this core self may lead to serious implications for the individual [1,3]. While all people perceive change in the self over time, there are individual differences in the degree of these changes [4]. Although a growing body of research examines perceptions of temporal self-continuity and its relationship to psychological outcomes, open empirical questions remain, and research has yet to understand the role of an individual’s background in these relationships.

The present research hypothesized that differences in individuals’ backgrounds—specifically, the extent of their financial resources (i.e., socioeconomic status)—may be instrumental to understanding (1) how continuous perceptions of the self are over time (i.e., past-to-present and present-to-future) and (2) the relationship between temporal self-perceptions and important psychological outcomes (i.e., mental health and well-being). Importantly, identifying these differences may provide crucial information for interventions designed to address persistent inequities in psychological well-being and health between socioeconomic groups [5,6,7,8].

Below, we begin with a brief review of the existing research on the continuity of temporal self-perceptions and the relationship between these perceptions and psychological outcomes (i.e., mental health and well-being). Within these reviews, we highlight the open empirical questions in the literature that the current research aims to address. We then present a rationale for (1) why we expect the continuity of temporal self-perceptions, and its relationship to psychological outcomes, to vary across socioeconomic status and (2) why college students present an effective starting point for investigating these relationships. Finally, we present the aims and hypotheses of the current research.

### 1.1. Continuity of Temporal Self-Perceptions

Across the lifespan, individuals undergo physical, environmental, and psychological changes. Despite these inevitable changes, theorists in both philosophy and psychology suggest that the self is comprised of not only present thoughts and emotions but also past memories and the anticipated future (e.g., [1,2,9,10]). As such, individuals should perceive a degree of continuity of the self across time. In fact, Erikson [1] portrayed the perception that the self is the same, or continuous, over time as an integral component of identity development. According to Erikson’s stages of psychosocial development, adolescents search for answers to questions such as “Who am I?” and “What do I want to become?” In this framework, individuals must construct a meaningful self-concept, uniting the past and present with future aspirations to establish a coherent identity that is stable across time, acting as a base upon which to determine realistic goals and attain personally meaningful objectives. The extent to which one perceives the self as a stable, continuous entity determines the degree of continuity of temporal self-perceptions, which encompasses two directional components: past-to-present and present-to-future [11].

Continuity of temporal self-perceptions and its implications are a growing body of research with many researchers focusing on continuity in one temporal direction (i.e., the degree of continuity between the past and the present or the present and the future). For example, the derailment literature focuses on the degree of perceived disconnect between the present self and who the individual used to be (i.e., a lack of continuity between the past and the present; [12,13]). Additionally, the literature on nostalgia for the past (i.e., “a sentimental longing for one’s past;” [14], p. 305), finds that nostalgia supports perceived continuity between the past and present self [15]. Similarly, many lines of research consider continuity between the present and the anticipated future self (e.g., future self-continuity [16]; psychological connectedness [17]; future self-identification [18]). These avenues of research have provided extensive evidence of the importance of both past-to-present and present-to-future self-continuity for a myriad of outcomes (e.g., intertemporal decision-making, self-control, academic success, mental health) but do not provide clarity on the extent or impact of continuity of temporal self-perceptions across time—past-to-present-to-future.

Importantly, recent studies are moving in the direction of considering a bi-directional assessment of continuity of temporal self-perceptions and its impact on psychological outcomes (e.g., [11,19,20]). This emerging literature has employed two measures of continuity across both temporal directions (i.e., the past and present self and the present and future self). The first measure is revised from the future self-continuity literature—originally based on the Inclusion of Other in the Self visual scale [21]—and asks study participants to select one of seven pairs of circles with varying degrees of overlap to indicate the degree of similarity/connectedness between (1) the past and present self and (2) the present and future self [4]. The second measure calculates participants’ perceptions of absolute change from past-to-present and present-to-future using a revised version of the me/not me task [22,23]. The task asks participants to rate how descriptive a list of positive (e.g., calm) and negative (e.g., tense) traits are of them in the past, present, and future. Based on these ratings, researchers calculate the degree of perceived change in the self from past-to-present and present-to-future. These measures evaluate the continuity of one aspect of temporal self-perception: the perception that the present self is similar/connected to the past and future self. In terms of similarity/connectedness between temporal selves, these studies suggest a moderate degree of continuity across time (i.e., bivariate correlations between past-to-present and present-to-future similarity were significant and ranged from 0.41 to 0.52); [19] with one study finding that the continuity of perceptions of temporal self-similarity/connectedness increased with the participants’ age [11]).

Although these prior studies focused on the continuity of similarity/connectedness over time, research and theorizing on perceptions of temporal selves suggests that there are three distinct factors that contribute uniquely to understanding how an individual perceives their temporal selves: (1) similarity and connectedness to the temporal self, (2) vividness of imagining the temporal self, and (3) positivity toward the temporal self [16,18,24]. To advance the literature in this area, the current research sought to extend the understanding of the continuity of temporal self-perceptions by taking into account the continuity of all three factors from past-to-present and present-to-future.

### 1.2. Continuity of Temporal Self-Perceptions, Mental Health, and Psychological Well-Being

A growing body of literature supports a relationship between the continuity of temporal self-perceptions and positive psychological outcomes (i.e., psychological well-being and mental health). Early work with psychiatric inpatients found that patients who had trouble constructing a continuous temporal self were more likely to be considered at high risk for suicide [3]. More recent research found an association between low levels of continuity and negative psychological outcomes (e.g., suicidal ideation, depressed mood; [19,20,25,26]). Additionally, higher levels of continuity are associated with engaging in adaptive behavior following a setback (i.e., losing a job [27]).

Despite the strong foundation in the literature for the relationship between continuity of temporal self-perceptions and positive psychological outcomes, the specific roles of past-to-present and present-to-future continuity remain ambiguous. For example, in a line of research aiming to assess discontinuity between the past and present self (i.e., derailment [12]), findings provide mixed results on the relationship between discontinuity and mental health (e.g., depression symptoms). The Derailment Scale [12] measures the extent to which one reports a discrepancy between the past self and the present self; those who report lower continuity between the past and the present selves are said to be “derailed.” If continuity with the past self is beneficial to mental health, derailment should be associated with higher levels of mental health difficulties such as depression symptoms. Some research supports this relationship, finding that individuals with greater depressive symptoms felt a stronger disconnect between their past and present [12,28]. However, additional longitudinal work on the derailment construct found that a disconnect between the past and present did not predict greater future depression symptoms [13].

Additionally, some lines of research that have considered both past-to-present and present-to-future continuity simultaneously suggest that continuity with the future self, and not the past self, may be the critical indicator of psychological outcomes and mental health [20,25]. Specifically, in studies of clinical populations, continuity from present-to-future emerged as the important predictor of mental illness, mood, and symptom severity [20,25,29]. In a non-clinical sample, continuity from present-to-future was associated with greater satisfaction with life while past-to-present continuity was not [30].

Relatedly, considering the relationship between experimentally increased continuity and psychological well-being (e.g., self-esteem, mood) following an impossible task, researchers found that the manipulation succeeded in increasing present-to-future, but not past-to-present, continuity [30]. Despite only increasing continuity in one temporal direction, the manipulation succeeded in buffering the experimental group participants’ self-esteem and mood following failure at the impossible task. This finding suggests that perceived continuity from present-to-future may be the critical aspect in supporting psychological well-being. Taken together, the existing literature suggests that it remains an open question whether there is a differential effect in how both directions of perceived continuity of the self (past-to-present and present-to-future) predict well-being and mental health. To address this need in the literature, the current research sought to understand if both past-to-present and present-to-future temporal self-perceptions contribute uniquely to predicting an individual’s mental health and well-being.

### 1.3. Socioeconomic Status and Continuity of Temporal Self-Perceptions

Importantly, the role of an individual’s background in continuity of temporal self-perceptions, and how that perceived continuity relates to their psychological outcomes, also remains unclear. While all individuals perceive some degree of change in the self over time, there are individual differences in the extent of these changes [4]. However, apart from work showing that continuity of temporal self-perceptions increases with age, little research has assessed individual differences in perceived continuity [11], nor the interaction between perceived continuity and an individual’s background when predicting psychological outcomes. Importantly, assessing the relationship between an individual’s background and continuity of temporal self-perceptions may provide insight into the inconsistent findings in past research.

The present research aimed to begin to expand this area of the literature by focusing on socioeconomic background. Building and extending on temporal self-appraisal theory [31,32], we propose that people from financially disadvantaged backgrounds (i.e., low socioeconomic status), as compared to individuals from high financial status backgrounds, may differ in their continuity of temporal self-perceptions. Temporal self-appraisal theory is based on the premise that individuals evaluate their past selves in a way that allows them to perceive their present selves more positively [31,32,33,34,35]. In experimental tests of temporal self-appraisal theory, the results suggest that individuals not only hold less positive perceptions of their past compared to their present selves, but they are also more likely to perceive a negative past as less connected to their present self [31,32]. This line of theorizing and research may provide insight into the continuity of temporal self-perceptions for those from financially disadvantaged pasts. For example, someone who had a difficult past (e.g., economic challenges) and now has a more economically stable present, and promising future, may be particularly likely to perceive their negative past as disconnected and discontinuous from their present and future selves. Additionally, they may interpret differences between past and present selves as the result of positive growth and therefore perceive the present and future self as continuous despite marked differences from the past to the present. For example, college students from low socioeconomic status backgrounds may perceive strong continuity from their present to their future self despite low perceived continuity with the past self. For these college students, the overall continuity of temporal self-perceptions, from past-to-present and present-to-future, may be less beneficial than the perceived trajectory of the self over time. In other words, for low socioeconomic status students, a strong and clear connection to a positive future self may be particularly important for supporting their mental health and psychological well-being. Importantly, socioeconomic background has a well-established relationship with an individual’s psychological outcomes (i.e., well-being and mental health). A meta-analysis of 357 studies assessing the relationship between socioeconomic status and subjective well-being found that both objective and subjective measures of socioeconomic status predict subjective well-being [8]. Perceived continuity between the temporal selves may be an adaptive strategy for bolstering psychological well-being for individuals from economically disadvantaged backgrounds.

### 1.4. College Students and Continuity of Temporal Self-Perceptions

To investigate the continuity of temporal selves and their relationship to socioeconomic status, the current research focused on college student populations. Young college students are an effective starting point for this research for three specific reasons: (1) College students are at a unique stage in their development. According to Erikson [1], students in their first years of college (i.e., 18-year-olds) are nearing the end of their “Identity vs. Confusion” stage of development. Throughout their adolescence, they explored who they were and the person they desired to be. This stage is characterized by searching and commitment and, optimally, results in an integrated self [36,37]. As such, young college students are at a developmental stage where we may expect to see a clear perception of the continuity of temporal selves and may provide a starting point to begin an investigation into the strength of the relationship between temporal selves. (2) College students are at a time in their lives when they are repeatedly presented with decisions that have important consequences that play out over time (i.e., intertemporal decisions). These intertemporal decisions may have a large impact on students’ long-term psychological well-being and mental health. The continuity of temporal self-perceptions is an important predictor of how individuals approach intertemporal decision-making (e.g., decisions to save money for the future and decreased academic procrastination [4,17,38]). Given the prevalence of intertemporal decisions in college students’ everyday lives, investigating the relationship between perceptions of temporal selves and psychological outcomes may be particularly important for this population. (3) A goal of higher education is to provide an avenue for social mobility. A college degree allows students from disadvantaged backgrounds to move beyond difficult aspects of their past (e.g., economic insecurity) and focus on positive opportunities for their future. As a result, a college student sample provides a critical test of the relationship between a disadvantaged background (i.e., low socioeconomic status) and the continuity of temporal self-perceptions.

### 1.5. Overview of the Research

The present research had three aims. Our first aim was to test the degree of continuity of temporal self-perceptions in college students. Specifically, we aimed to (a) determine if perception of the temporal selves (i.e., from past-to-present and present-to-future) was continuous and (b) further probe that perceived continuity to test if it varied across the three factors of temporal self-perception. In line with previous research, we hypothesized that perception of the past self and the future self would be moderately, but not strongly, correlated. To test this first aim, we addressed the following research questions: (RQ1a) What was the relationship between perceived past and future selves?; and (RQ1b) Did this relationship vary across the three factors of temporal self-perceptions (similarity/connectedness, vividness, positivity)?

Our second aim was to investigate the extent to which perceptions of the past self and future self predicted mental health and psychological well-being. We hypothesized that perception of the past self and perception of the future self would both be critical predictors of an individual’s mental health and well-being. Specifically, we asked: (RQ2) Did perception of the past self and the future self jointly and independently predict depression symptoms and psychological well-being (i.e., meaning in life, life satisfaction)?

Our third aim was to explore whether the perceived continuity of temporal selves varied across socioeconomic groups. In comparison to individuals with greater economic resources, we expected students from financially disadvantaged backgrounds (i.e., lower socioeconomic status in terms of household income) to differ in continuity of temporal self-perceptions. Specifically, we hypothesized that the perception of the past self would be less predictive of the future self for students from low SES families than for their counterparts from higher SES families (i.e., low SES students would report greater discontinuity between the past and the present than high SES students). Additionally, we aimed to explore if the relationship between perceived continuity of temporal selves and mental health and psychological outcomes differed across SES groups. To address this final aim, we explored the final research question: (RQ3) Did continuity of temporal self-perceptions (i.e., the relationship between past and future self-perceptions), and its relationship with mental health and psychological outcomes, differ by socioeconomic background?

## 2. Materials and Methods

### 2.1. Design, Setting, and Participants

This research used a cross-sectional design with two independent samples. We recruited students from Introduction to Psychology courses at a large, public university in the southwestern United States where students participated in research to earn course credit. The research reported here used that psychology participant pool to collect two samples to (1) test and (2) replicate and extend our findings. Sample 1 (*N* = 391) was collected at the start of the Spring 2020 semester and Sample 2 (*N* = 257) was collected mid-semester. Sample 1 data collection took place from 21 to 30 January 2020. This was prior to the initial spread of COVID-19 in the area. Sample 2 data collection took place from 24 to 25 March 2020. This was during the initial spread of COVID-19 (i.e., the university had announced a move to online courses the previous week). The impact of COVID-19 was not the a priori focus of this research. Additionally, we did not expect a significant impact on the continuity of temporal self-perceptions at the early stage of the pandemic. As such, we do not focus on it here. However, when possible, we do replicate the findings across the two samples to lend support to the conclusion that our results were not dependent on the global pandemic.

Both samples were recruited online through SONA Systems, were similar in age (Sample 1: *M* = 19.03, *SD* = 1.43; Sample 2: *M* = 19.24, *SD* = 1.54), gender distribution (Sample 1: 56% female, Sample 2: 60% female), and ethnic diversity (Sample 1: 51.5% White, 16.4% Asian, 13.6% Hispanic/Latino, 3.3% Black, 1.3% Middle Eastern, 0.8% Native American, and 13.1% more than one race indicated; Sample 2: 41% White, 22.3% Asian, 10.9% Hispanic/Latino, 3.5% Black, 2.3% Middle Eastern, 0.8% Native American, 0.8% Other, and 18.4% more than one race indicated). Sample 1 was collected as a part of a larger, department-wide prescreening questionnaire and Sample 2 was collected as a stand-alone study. Participants who participated in both Sample 1 and Sample 2 (*n* = 43) were excluded from the Sample 2 analyses to prevent counting the same participant twice and repeated administration effects.

### 2.2. Variables and Measurement

#### 2.2.1. Perception of the Future Self

We measured the perception of the future self using the 6-item Future Self-Identification Scale [18]. This scale includes two items to measure each of the three factors of how the present self perceives the future self (i.e., similarity/connectedness (also known as relatedness [18], vividness, and positivity). The participants were first instructed to think of themselves five years in the future. This time period corresponds to a future point when many college graduates are establishing their careers and families. They then completed the six items. Example items include:

Future Self-Similarity/Connectedness: “Please select how similar you feel to your future self five years from today? 1 = not at all similar to my future self, 7 = very similar to my future self”

Future Self-Vividness: “When you imagine your future self, how vividly do you picture it? 1 = not at all vividly; I do not have a clear image in my head of my future self, 7 = very vividly; I have a very clear image in my head of my future self”

Future Self-Positivity: “Please characterize your future: 1 = very negative, 7 = very positive.”

All items were measured on a 7-point scale. Past research demonstrated that perception of the future self was a significant predictor of greater self-control, psychological well-being, and academic performance [18].

We created an aggregate of the full scale (6 items) as well as aggregates for each of the three factors (2 items each). Henceforth, for ease of communication, we refer to the full-scale aggregate as the perception of the future self and the individual factors as future self-similarity/connectedness, future self-positivity, and future self-vividness. In both samples, the reliability of the full scale and the factor scales were acceptable (Sample 1 *α*’s: Perception of the Future Self = 0.78; Future Self-Similarity/Connectedness = 0.69; Future Self-Positivity = 0.75; Future Self-Vividness = 0.90; Sample 2 *α*’s: Perception of the Future Self = 0.80; Future Self-Similarity/Connectedness = 0.75; Future Self-Positivity = 0.74; Future Self-Vividness = 0.90).

#### 2.2.2. Perception of the Past Self

To measure how the present self perceives the past self, we revised the 6-item Future Self-Identification Scale [18]. We revised all mentions of the future to read the past. The participants were instructed to think of themselves five years in the past. Beyond this change, the items and response options were identical to the perception of the future self measure described above. Again, we created an aggregate for the full scale and for each of the three factors. As above, henceforth we refer to the full-scale aggregate as perception of the past self and the individual factors as past self-similarity/connectedness, past self-positivity, and past self-vividness. In both samples, the reliability of the full scale and the factor scales were acceptable (Sample 1 *α*’s: Perception of the Past Self = 0.69; Past Self-Similarity/Connectedness = 0.71; Past Self-Positivity = 0.79; Past Self-Vividness = 0.88; Sample 2 *α*’s: Perception of the Past Self = 0.74; Past Self-Similarity/Connectedness = 0.77; Past Self-Positivity = 0.76; Past Self-Vividness = 0.85).

#### 2.2.3. Depression Symptoms

We measured depression symptoms using the Center for Epidemiological Studies Depression scale (CES-D; [39]. This 20-item scale measures symptoms of depression in the past week (e.g., “I felt fearful;” “My sleep was restless”). Participants rated the items on a 4-point scale (0 = Rarely or none of the time (less than 1 day); 3 = All of the time (5–7 days)). We created an aggregate depression symptom score by taking the sum of the 20 items. Scores could range from 0 to 60. In the literature, a sum score of 16 points or greater is indicative of depression. Reliability for the scale was excellent in both samples (Sample 1: *α* = 0.90; Sample 2: *α* = 0.90).

#### 2.2.4. Psychological Well-Being

Both measures of psychological well-being were collected only in Sample 2.

Meaning in Life: Presence. To measure the presence of meaning in life, we used the 5-item presence subscale from the Meaning in Life Scale (e.g., “I understand my life’s meaning”, [40]. Participants rated items on a 7-point scale (1 = Absolutely untrue; 7 = Absolutely true). High scores on this scale are associated with greater positive affect, extraversion, and agreeableness, and lower negative affect, depression, and neuroticism. The reliability of the scale was good (Sample 2: *α* = 0.85).

Satisfaction with Life. As a measure of subjective well-being, we used the 5-item Satisfaction with Life Scale (e.g., “In most ways my life is close to ideal; [41]). Items were rated on a 7-point response scale (1 = Strongly disagree, 7 = Strongly agree). High satisfaction with life scores predict greater self-esteem, positive affect, and optimism and lower negative affect, depression, and suicidal ideation (for a review see [42]). The scale demonstrated very good reliability (Sample 2: *α* = 0.88).

#### 2.2.5. Socioeconomic Status

To assess socioeconomic status, participants completed one item: “In terms of income, how would you describe you and your family’s socio-economic status?” The five response options included working class (Sample 1: *n* = 28; Sample 2: *n* = 11), lower-middle class (Sample 1: *n* = 52; Sample 2: *n* = 30), middle class (Sample 1: *n* = 152; Sample 2: *n* = 118), upper-middle class (Sample 1: *n* = 142; Sample 2: *n* = 87), upper class (Sample 1: *n* = 12; Sample 2: *n* = 7), and other, please specify (Sample 1: *n* = 1; Sample 2: *n* = 3). We excluded participants who selected other from the socioeconomic status analyses as their responses did not allow for a clear determination of status (e.g., “Prefer not to share”). Five participants did not respond to the socioeconomic status item (Sample 1: *n* = 4; Sample 2: *n* = 1). The aim of this research was to assess differences in temporal self-continuity in students across levels of economic security. Specifically, we hypothesized differences in the continuity of temporal self-perceptions between students who were economically disadvantaged (i.e., working class, lower-middle class) and those with relatively high economic resources (i.e., upper-middle class; upper class). For students with average economic security (i.e., middle class), we sought to explore if their continuity of temporal self-perceptions mirrored that of either their higher or lower socioeconomic counterparts or if their continuity fell between that of the two economic extremes. As such, we created a three-category socioeconomic status variable (1 = Below middle class; 2 = Middle class; 3 = Above middle class).

#### 2.2.6. Parental Educational Attainment

An important factor in the conclusions of this research is the meaning of our socioeconomic measure. In other words, is a student’s report of their family’s socioeconomic status related to the relative financial security of their family? To address this issue, we tested the relationship between our SES measure and parental (mother and father) educational attainment. Specifically, the two parental education items asked the students to indicate the highest level of education attained by their mother and father respectively: (1) less than high school, (2) high school diploma (or GED), (3) some college or a 2-year college degree, (4) 4-year college degree (B.A., B.S.), (5) Master’s degree (M.A., M.S.), (6) Graduate or professional degree (J.D., Ph.D., M.D.), (7) other (please specify). Students who indicated “other” were removed from the analyses unless their entry under “please specify” allowed them to be clearly placed into one of the six groups.

### 2.3. Procedure

Sample 1 was collected as part of a larger prescreening questionnaire that was administered to Introduction to Psychology students at the target university. Sample 2 participants were recruited through a study posting in SONA Systems. Both samples completed the survey online through the Qualtrics platform. Students first read a consent form and indicated that they were at least 18 years of age. Clicking the next button on the survey indicated their consent to participate. The measures of perceptions of the future and past selves were presented in randomized order. Following the perception of the future and past selves scales, students completed the CES-D (Sample 1 and 2), the Satisfaction with Life Scale (Sample 2), and the Meaning in Life Scale (Sample 2). As Sample 1 was collected as a part of a prescreening survey, those participants completed demographic items (e.g., sex, socioeconomic status) at the start of the survey. Sample 2 completed the same demographic items at the end of the survey. Sample 1’s prescreening questionnaire took approximately 60 min to complete. The survey for Sample 2 took approximately 30 min to complete. After completing the survey, students read a debriefing message and received course credit.

### 2.4. Statistical Methods and Study Size

For each hypothesis detailed above, we first tested the hypothesis in Sample 1 using bivariate correlations and OLS regression and then replicated the findings in Sample 2. Additionally, Sample 2 allowed us to extend our results for Research Question 2 to test the relationship between past and future self-perceptions and measures of psychological well-being (i.e., meaning in life and life satisfaction). We conducted all analyses using SPSS (V.25) and PROCESS (V.3.3; [43]). The effect sizes in this research are evaluated using the following benchmarks: *r* = 0.10 (small/weak), *r* = 0.20 (medium/moderate), and *r* = 0.30 (large/strong) [44].

We conducted a series of power analyses using G*Power (3.1.9.4) to determine the necessary sample size to test our hypotheses. Assuming a medium effect size (i.e., *f*^2^ = 0.15), and a 0.05 two-tailed significance value, our most complex planned analyses (e.g., a two-way interaction between socioeconomic status and perception of the future self) required a minimum total sample size of 77 to achieve 0.80 power. However, a major aim of this research was to understand differences in the continuity of temporal self-perceptions by socioeconomic status. As such, we oversampled to ensure a sufficient number of students from different socioeconomic backgrounds across our samples.

## 3. Results

### 3.1. Descriptive Data

In Sample 1, 20 percent of the students identified as below the middle class, 39 percent as middle class, and 41 percent as above the middle class. Similarly, in Sample 2, 16 percent identified as below the middle class, 47 percent as middle class, and 37 percent as above the middle class. In both samples, students reported socioeconomic status was positively correlated with higher educational attainment by both mothers and fathers (SES and Mother’s Education: Sample 1: *r*(381) = 0.40, *p* < 0.001; Sample 2: *r*(253) = 0.33, *p* < 0.001; SES and Father’s Education: Sample 1: *r*(380) = 0.43, *p* < 0.001; Sample 2: *r*(250) = 0.36, *p* < 0.001). See the Supplementary Materials for crosstabulation tables showing the distribution of parental education levels by self-reported socioeconomic status. These results suggest that the student self-report socioeconomic status measure is positively associated with parental educational attainment (i.e., a proxy for financial resources).

Table 1 presents the descriptive statistics by sample and by socioeconomic status for the relevant study variables. Overall, participants’ perception of the future self was above the mid-point on the 7-point scale. This finding held for the aggregate perception of the future self as well as for each of the three factors (i.e., similarity/connectedness, positivity, vividness). Across the samples, positivity toward the future self was well above the mid-point. These findings held regardless of socioeconomic status suggesting that, on average, students who reported relatively strong perceptions of their future selves and especially positive toward their future selves. In terms of the past self, perception of the past self, past positivity, and past vividness, or at, the mid-point on the 7-point scale. The perceived vividness of the past self was particularly high, suggesting that, on average, students could clearly and vividly image their past (*Ms* ranged from 4.77 to 5.18). In contrast, similarity/connectedness to the past self was below the mid-point across the samples and levels of socioeconomic status. This trend suggests that, on average, students felt relatively disconnected and dissimilar to their past selves. Considering depressive symptoms, across the samples, on average, participants were at or above the 16-point cutoff indicating clinical depression (*Ms* ranged from 16.33 to 21.29) but below the midpoint on the 60-point depression symptom scale. In terms of psychological well-being, across the samples, well-being ratings (i.e., satisfaction with life and meaning in life) were above the mid-point on their 7-point scales. These findings suggest that overall, the students experienced moderate (i.e., slightly above the cutoff for depression) depression symptoms and psychological well-being.

Table 2 presents the bivariate correlations among the variables of interest. Similar to previous findings, the perception of the future self factors were positively correlated (*rs* ranged from 0.30 to 0.48; [18]). Similarly, past self-similarity/connectedness was positively correlated with positivity toward the past self (Sample 1: *r*(391) = 0.46; Sample 2: *r*(257) = 0.48). However, past vividness was only moderately, or in one case insignificantly, related to past self-similarity/connectedness and past positivity (*rs* ranged from 0.05 to 0.21). Given the high scores on past vividness on average, this may suggest that students were able to vividly image their past self regardless of their connection or positive feelings toward that self. On the whole, high ratings of perceptions of the future and past selves predicted lower depression symptoms and greater psychological well-being. The relationship between perceptions of the future and past self is detailed in the results for Research Question 1 below.

Below, we present the results for each of the research questions in turn. Where applicable, we first detail the results for Sample 1 and then provide the replicated results for Sample 2.

**Table 2:** Correlations among study variables.

	1	2	3	4	5	6	7	8	9	10	11
1. Perception of Future Self		0.75 **	0.75 **	0.82 **	0.29 **	0.20 **	0.19 **	0.25 **	−0.34 **	0.43 **	0.42 **
2. Future Similarity/Conn.	0.74 **		0.38 **	0.36 **	0.34 **	0.35 **	0.22 **	0.18 **	−0.29 **	0.35 **	0.25 **
3. Future Positivity	0.69 **	0.30 **		0.48 **	0.14 *	0.03	0.12	0.16 **	−0.32 **	0.42 **	0.41 **
4. Future Vividness	0.84 **	0.37 **	0.44 **		0.18 **	0.08	0.09	0.22 **	−0.20 **	0.25 **	0.34 **
5. Perception of Past Self	0.21 **	0.23 **	0.15 **	0.11 *		0.76 **	0.77 **	0.65 **	−0.31 **	0.28 **	0.24 **
6. Past Similarity/Conn.	0.19 **	0.30 **	0.08	0.05	0.76 **		0.48 **	0.21 **	−0.25 **	0.23 **	0.17 **
7. Past Positivity	0.12 *	0.17 **	0.08	0.30	0.74 **	0.46 **		0.19 **	−0.25 **	0.24 **	0.14 *
8. Past Vividness	0.14 **	0.03	0.14 **	0.15 **	0.58 **	0.15 **	0.05		−0.18 **	0.13 *	0.22 **
9. Depression	−0.32 **	−0.28 **	−0.27 **	−0.19 **	−0.18 **	−0.12 *	−0.19 **	−0.06		−0.53 **	−0.40 **
10. Satisfaction with Life	--	--	--	--	--	--	--	--	--		0.63 **
11. Meaning in Life	--	--	--	--	--	--	--	--	--	--	

Note. Sample 1 = Below the diagonal. Sample 2 = Above the diagonal. ** *p* < 0.01, * *p* < 0.05.

### 3.2. Research Question 1a. What Was the Relationship between Perceived Past and Future Selves?

As hypothesized, considering the full-scale measures of self-perception, students’ perceptions of their future selves and their past selves were moderately related constructs. In Sample 1, students’ ratings of perceptions of the past self were moderately and positively correlated with their perception of their future self, *r*(391) = 0.21, *p* < 0.001. Higher ratings of perceptions of the past self were indicative of higher ratings of perceptions of the future self. Replicating this result in Sample 2, we found a similar relationship in both direction and magnitude, *r*(257) = 0.29, *p* < 0.001. Importantly, the difference in magnitude in the correlation between the two samples was not significant suggesting a similar relationship across samples, *z* = −1.06, *p* = 0.289.

### 3.3. Research Question 1b. Did This Relationship Vary across the Three Aspects of Continuity of Temporal Self-Perceptions (Similarity/Connectedness, Vividness, Positivity)?

Perceptions of similarity/connectedness and vividness were continuous from past to future. Specifically, similarity/connectedness to the past self was strongly and positively related to future self-similarity/connectedness, *r*(391) = 0.30, *p* < 0.001. Vividness of the past self was weakly and positively related to future vividness, *r*(391) = 0.15, *p* = 0.002. Contrastingly, perceptions of positivity toward the past self were not significantly related to future self-positivity, *r*(391) = 0.08, *p* = 0.124. Within Sample 1, we assessed if there were significant differences in continuity between the factors (i.e., significant differences in the strength of the correlations between past and future self-perception). The continuity of similarity/connectedness was significantly greater than both the continuity of vividness (*z* = 2.21, *p* = 0.027) and positivity, *z* = 3.19, *p* = 0.001. The continuity of vividness did not significantly differ from the continuity of positivity, *z* = 0.99, *p* = 0.322.

Replicating the results in Sample 2, we found the same pattern of relationships between the continuity of the factors from past to future (Similarity/Connectedness: *r*(257) = 0.35, *p* < 0.001; Vividness: *r*(257) = 0.22, *p* < 0.001; Positivity: *r*(257) = 0.12, *p* = 0.061). The correlations in Sample 2 did not differ significantly from those in Sample 1 (Similarity/Connectedness: *z* = −0.69, *p* = 0.490; Vividness: *z* = −0.90, *p* = 0.368; Positivity: *z* = −0.50, *p* = 0.617). Assessing if there were significant differences in continuity between the factors, we found a similar pattern of results. The continuity of similarity/connectedness was significantly greater than the continuity of positivity (*z* = 2.76, *p* = 0.006) and trending in that direction for vividness, *z* = 1.6, *p* = 0.110. Again, the continuity of vividness did not significantly differ from the continuity of positivity, *z* = 1.16, *p* = 0.246.

Taken together, these results suggest that the continuity of temporal self-perceptions differed across the factors (connectedness/similarity, vividness, and positivity). Students with a strong perception of similarity and connection to their past selves were more likely to have a strong connection to their future selves. A vivid view of the temporal self was only weakly continuous suggesting that a vivid view of the past self only weakly predicted a vivid view of the future self. Positivity toward the past self was not predictive of positivity toward the future self. In other words, positivity was relatively discontinuous.

### 3.4. Research Question 2. Did Perception of the Past Self and the Future Self Jointly and Independently Predict Depressive Symptoms and Psychological Well-Being (i.e., Meaning in Life and Life Satisfaction)?

Table 3 provides the full regression results by psychological outcome. As hypothesized, perception of the past self and perception of the future self jointly and independently predicted each of our psychological outcomes. Students with higher ratings of perceptions of their past and future selves were less likely to have symptoms of depression and more likely to feel satisfaction and meaning in their lives. For the depression outcome, we replicated that result in Sample 2. Taken together, the results indicated that perceptions of both the past and future self were both important predictors of psychological well-being and mental health.

### 3.5. Research Question 3. Did Continuity of Temporal Self-Perceptions (i.e., the Relationship between Perceptions of Past and Future Selves), and Its Relationship with Mental Health and Psychological Outcomes, Differ by Socioeconomic Background?

#### 3.5.1. Continuity of Temporal Self-Perceptions by Socioeconomic Status

To test if the relationships between perceptions of the past and future selves, and each of the factors, held regardless of socioeconomic status, we conducted a series of moderation analyses. As the socioeconomic status variable was a multi-categorical variable with three categories (low, middle, and high), we used indicator coding where low socioeconomic status was set as the indicator. As a result, for each of these multi-categorical moderation analyses, there were three results that provided insight into the impact of socioeconomic status on continuity of temporal self-perceptions: (1) the significance of the interaction for students from low versus middle SES; (2) the significance of the interaction for students from low versus high SES; and (3) the significance of the additional variance explained by including the overall interaction (i.e., two interaction terms) in the model (i.e., *R*^2^ change).

**Figure 1:**
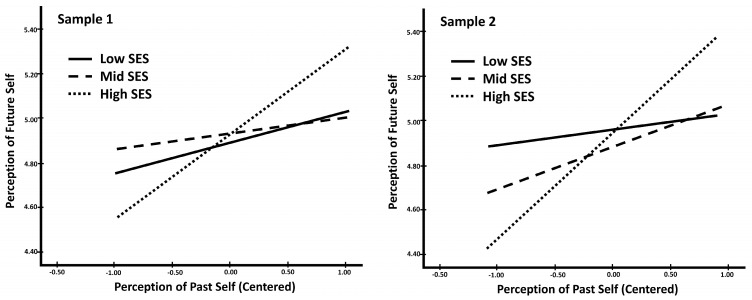
Interaction between perception of the past self and socioeconomic status predicting perception of the future self.

First, to understand the relationship between socioeconomic status and the overall continuity of temporal self-perceptions, we tested if socioeconomic status moderated the relationship between the perception of the past self and the perception of the future self. Specifically, we tested if perception of the past, socioeconomic status, and their interactions predicted levels of perception of the future self (see Table 4 for the full regression results). The interaction between perceptions of the past self and low versus high socioeconomic status was marginally significant in Sample 1 (*p* = 0.089) and significant in Sample 2 (*p* = 0.019), while the interaction with low SES versus middle SES was not significant in either sample (Sample 1: *p* = 0.658; Sample 2: *p* = 0.496). Including the overall interaction between perception of the past self and socioeconomic status in the regression equation led to a significant increase in the variance in perception of the future self explained by the model in both samples (see Table 4). In both samples, the interaction demonstrated the same trend (see Figure 1). The strength of the relationship between perception of the past self and perception of the future self (i.e., the continuity of temporal self-perception), varied as a function of a student’s socioeconomic status. Specifically, in both samples, for students with high socioeconomic status, a stronger perception of the past self was a clear indicator of a stronger perception of the future self. This was not the case for low socioeconomic status students. In both samples, students from the lower SES group did not significantly differ from their middle SES counterparts.

Additionally, based on the simple slopes, for low socioeconomic status students, perception of the past self was not a significant predictor of perception of the future self (see Table 5). Contrastingly, for high socioeconomic status students, a higher reported perception of the past self predicted a higher reported perception of the future self. Taken together, these results suggest that, compared to higher socioeconomic status students, continuity of temporal self-perceptions did not hold for students from lower socioeconomic backgrounds. The simple slope analyses for middle-class students led to a difference between the two samples where, in Sample 1, perception of the past was not a significant predictor of perception of the future. We will explore the differences in these findings in the discussion section below.

**Figure 2:**
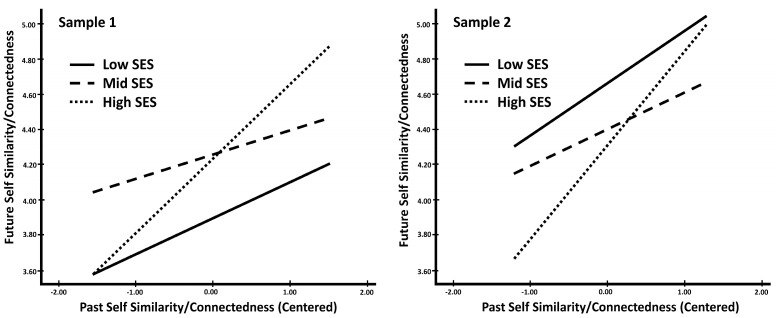
Interaction between past self-similarity/connectedness and SES predicting future self-similarity/connectedness.

To further understand the nature of this difference in continuity by socioeconomic status, we tested the moderation for each factor (i.e., similarity/connectedness, vividness, positivity) separately. The similarity/connectedness analyses most closely mirrored the results for overall continuity of temporal self-perceptions. In both samples, neither of the interactions between perceived similarity/connectedness to the past self and socioeconomic status (low versus middle; low versus high) reached significance when predicting perceived similarity/connectedness of the future self (see Table 6). However, in both samples, the overall interaction added significantly to the variance explained by the model (see Table 6 and Figure 2). The simple slope analyses showed that for high socioeconomic status students in both samples, higher perceptions of similarity/connectedness of the past self significantly predicted higher perceptions of future self-similarity/connectedness (see Table 7). For students from middle-status backgrounds, in Sample 1, the relationship was marginal and in Sample 2, it was significant (see Table 7). For low socioeconomic status students, similarity/connectedness to the past self was a marginal predictor of future similarity connectedness in both samples (see Table 7). Overall, the trend in Figure 2 suggests support for our hypothesis that temporal continuity between perceived similarity/connectedness would differ by socioeconomic status. The results suggest that the relationship between perceived similarity/connectedness to the past self and the future self was strongest for economically advantaged students (i.e., high socioeconomic status).

Considering the vividness factor, we observed a different trend. The interaction between the vividness of the past self and socioeconomic status was not a significant predictor of future self-vividness (see Table 8). The overall interaction did not significantly increase the variance explained by the model. The vividness results were contrary to our hypothesis, suggesting that, regardless of the student’s socioeconomic status, the vividness of the self demonstrated similar levels of continuity. We will return to this finding in the discussion section below.

Testing the interaction between socioeconomic status and past self-positivity as a predictor of future self-positivity, we observed varied results between samples. Specifically, in Sample 1, contrary to our hypothesis, the interactions were not significant predictors of future self-positivity (see Table 9). The overall interaction did not significantly increase the variance explained by the model. However, in Sample 2, we found support for our hypothesis; for low versus high SES students, the interaction between past self-positivity and socioeconomic status was significant. There was not a significant interaction for low versus middle socioeconomic status students. Additionally, in Sample 2, the overall interaction significantly increased the variance explained by the model.

**Figure 3:**
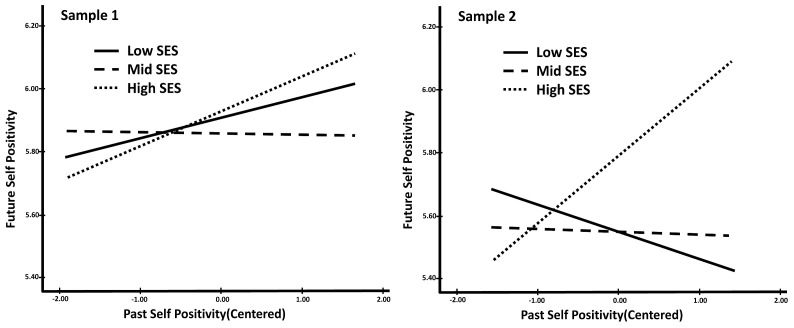
Interaction between past self-positivity and SES predicting future self-positivity.

The interactions for both samples display similar trends (see Figure 3). In fact, based on the simple slope analyses, both samples showed support for our hypothesis that students with lower economic resources would have less continuity in temporal positivity than higher SES students. In both samples, for low and middle SES students, past self-positivity did not predict future positivity, while that relationship was significant for high-status students (see Table 10). However, the magnitude of differences between socioeconomic groups was augmented in Sample 2 leading to the significant interaction result. In Sample 2, compared to economically disadvantaged students (i.e., low socioeconomic status), students from high socioeconomic backgrounds with high levels of past self-positivity were more likely to perceive their future selves positively. This difference between samples may be due to the timing of sampling (i.e., before the spread of the global COVID-19 pandemic (Sample 1) and after the initial spread (Sample 2)). We will return to this possibility in the discussion section.

#### 3.5.2. Temporal Self-Perceptions and Psychological Outcomes by Socioeconomic Status

For each psychological outcome (i.e., depression, satisfaction with life, and meaning in life), we ran separate regressions testing (1) perception of the past self, socioeconomic status, and their interactions predicting the outcome and (2) perception of the future self, socioeconomic status, and their interactions predicting the outcome. The interactions were not significant in predicting depression or satisfaction with life (*ps* ranged from 0.124 to 0.934; see Tables S1 and S2 in the Supplementary Materials for the regression results). In other words, regardless of the students’ socioeconomic status, both perceptions of the past self and the future self were significant, and additive, predictors of depression and life satisfaction.

**Figure 4:**
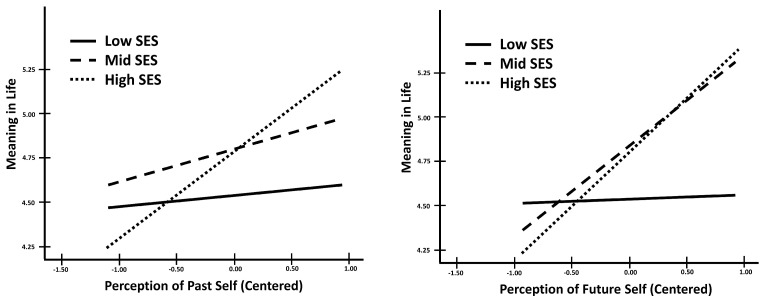
Interactions between temporal self-perceptions and SES predicting meaning in life. Perception of the past self is on the left and perception of the future self is on the right.

In terms of perceived meaning in life, for both perception of the past self and the future self, socioeconomic status was a significant moderator for low versus high SES students (see Table 11). For low versus middle SES students, the interaction between perception of the past self and socioeconomic status was insignificant in predicting meaning in life while the interaction between perception of the future self and status was marginal, *p* = 0.059. Additionally, in both the perception of the past and future self analyses, the increase in the variance explained by including the overall interaction in the model was marginal (see Table 11). Both interactions showed a similar trend (see Figure 4). Based on the simple slopes, for students from low socioeconomic backgrounds, neither perception of the past self nor the future self was a significant predictor of meaning in life (see Table 12). In contrast, for students from high socioeconomic groups, both perception of the past and the future self significantly predicted meaning in life. For middle-status students, perception of the past self did not significantly predict meaning in life (*p* = 0.142) but perception of the future did, *p* < 0.001. Compared to disadvantaged students, for students from higher socioeconomic status, higher reports of temporal self-perceptions, both past and future, predicted greater meaning in life. Predicting meaning in life from the perception of the past self, middle-status students fell between the two economic extremes. However, considering the relationship between perception of the future self and meaning in life, middle-status students closely matched their high-status counterparts. The finding that neither past nor future self-perceptions predict meaning in life for disadvantaged students was unexpected. We explore this finding in the discussion section.

## 4. Discussion

This research aimed to extend the literature on temporal self-perceptions in three areas. First, we aimed to enhance the understanding of the continuity of temporal self-perceptions by assessing all three hypothesized factors (i.e., similarity/connectedness, vividness, and positivity) across the two temporal directions (i.e., past-to-present and present-to-future). In line with our hypothesis, greater continuity between the past and present self was moderately indicative of greater continuity between the present and future self. Additionally, extending the literature to consider the continuity of the three factors separately (i.e., similarity/connectedness, vividness, and positivity), we found that the similarity/connectedness and vividness factors were both continuous across time. However, this relationship did not hold for positivity. Students who perceived their past selves positively were not more likely to report positive perceptions of their future selves. The lack of significant continuity of positivity across time may, in part, be related to temporal self-appraisal theory. The foundation of temporal self-appraisal suggests that perceptions of the past self are shaped to favor a positive perception of the present and future. In other words, students may alter their perceptions of their past self-positivity to contribute to greater positive perceptions of their present and future selves. Overall, our continuity findings were replicated across our two samples and support prior research suggesting moderate continuity of similarity between the past-to-present and the present-to-future selves [19]. These results provide evidence suggesting that perceptions of similarity/connectedness and vividness may be the crucial factors in maintaining even moderate continuity of temporal self-perceptions across time.

Our second aim was to test the relationships between both directions of temporal self-perception—taking into account the three proposed factors—and psychological outcomes (i.e., depression symptoms and psychological well-being). In support of our hypothesis, perceptions of the past self and the future self jointly and independently predicted less severity of depression symptoms and greater overall satisfaction and meaning in life. These results support theoretical conceptions that perceived continuity of the self across time (past-present-future) is important for maintaining mental health and well-being. However, our finding that both directions of temporal self-perceptions were important, unique predictors of psychological outcomes may add nuance to the prior understanding of the different directions of temporal self-continuity and their relationship with well-being. Specifically, recent studies suggested that present-to-future continuity, and not past-to-present continuity, was the stronger predictor of mental health and well-being [20,25,29,30]. The difference in these findings may be explained by differences in the measures of continuity across studies. The past research primarily focused on continuity in terms of similarity and connectedness of the temporal self across time, while our research expanded this measure to include the three hypothesized factors of temporal self-perception (i.e., similarity/connectedness, positivity, and vividness). The findings across our two samples suggest that when assessing the more comprehensive, three-factor temporal self-perception, both temporal directions contribute to positive psychological outcomes.

Our third and final aim was to determine if there were differences in temporal self-continuity, and its relationship to psychological outcomes, across socioeconomic backgrounds (i.e., low versus middle versus high). We first focus our discussion on the findings comparing students from the two economic extremes (i.e., low and high status) and how they relate to our hypothesis and then discuss the exploratory middle-class background results. First, we assessed if the relationship between perceptions of the past and future self differed by economic background. Supporting our hypothesis, for high socioeconomic status students, higher ratings of perceptions of the past self predicted higher ratings of perceptions of the future self. This was not the case for students from low socioeconomic status backgrounds. Overall, the results suggest that for students from challenging financial backgrounds, having high continuity with that difficult financial past may not be crucial to continuity with their future self.

To better understand this difference in continuity between students of low and high socioeconomic backgrounds, we also tested for differences in continuity for each of the three temporal self-perception factors separately (i.e., similarity/connectedness, vividness, and positivity). Similar to the findings for the overall temporal self-perception aggregate measures, the relationship between continuity of past self-similarity/connectedness and future self-similarity/connectedness was strongest for high SES students. Compared to low socioeconomic status students, high SES students who perceived greater similarity from past-to-present were more likely to perceive similarity from present-to-future. This was not the case for the vividness factor where the relationship between past self-vividness and future self-vividness was similar regardless of economic background. Taken together, these findings suggest that continuity between similarity/connectedness may be driving the differences in temporal self-continuity by socioeconomic status.

In terms of positive perceptions of the temporal selves, we found somewhat different results across our two samples. Sample 1 did not show a significant difference in continuity of positivity between low and high economic status while Sample 2 did. After assessing the interactions in the two samples, we found that students from high socioeconomic backgrounds with high levels of positive perceptions toward the past self were more likely to perceive their future self positively while this was not the case for those from economically disadvantaged backgrounds. Overall, the trend of the interaction in the two samples was the same although the magnitude of the difference by socioeconomic status was larger in the second sample. The difference in magnitude may be due to the timing of the sampling. Sample 1 was collected prior to the global spread of COVID-19 (i.e., January 2020) while Sample 2 was collected soon after COVID-19 became widespread (i.e., March 2020). Given the economic challenges presented by COVID-19 (e.g., shutdowns and job losses), especially for students who already suffered from a difficult economic background, the spread of the pandemic may have accentuated the difference in positivity continuity by socioeconomic status.

Considering students from middle socioeconomic status backgrounds, we aimed to explore if these students more closely mirrored their high or low SES counterparts’ temporal self-continuity. We did not find a significant difference between low and middle SES students in either sample. However, for middle SES students in Sample 1, we found that the perception of the past was not a significant predictor of the perception of the future. In Sample 2, the perception of the past self significantly predicted the perception of the future self for middle SES students. As such, the results from Sample 1 suggest that middle SES students more closely mirrored the temporal self-continuity of low SES students (i.e., their perception of the past self did not predict their perception of the future self), while Sample 2 suggests that these middle SES students were more similar to high SES students (i.e., their perceptions of their temporal selves were continuous from past to future). A possible explanation for this interesting difference between the samples may be a result of the onset of the COVID-19 pandemic. Specifically, prior to the pandemic, the continuity for higher SES students differed from both the low and middle SES students. However, when faced with the initial spread of the pandemic, and the economic uncertainty it presented, middle SES students’ continuity began to look more like that of students from higher SES backgrounds. During this uncertain time, middle SES students may have leaned more on their perception of their relatively stable past self in their perception of the newly uncertain future. As a result, after the onset of the pandemic, lower SES students showed the most unique pattern of continuity between their temporal self-perceptions (i.e., the relationship between their past and future self-perceptions remained discontinuous). This may indicate that lower SES students are more likely to pick up the tab from the many difficulties presented by the pandemic. Future studies should explore this possibility.

Importantly, a large portion of the United States population considers themselves to be middle class [45]. This portion may be even higher in samples of college students such as ours. However, while many self-report as middle class, the percentage of individuals who actually belong to the economic middle class is shrinking over time. This issue may be exacerbated by global challenges such as the COVID-19 pandemic. Additionally, in contrast to individuals from lower SES backgrounds, the middle class is not often the focus of interventions designed to enhance well-being and mental health. Future research may benefit from focusing on understanding factors that influence the continuity of temporal self-perceptions for students from the middle class.

In terms of socioeconomic status, our research focused on determining if the continuity of temporal selves varied across economic groups. Given our findings, future research may wish to broaden the understanding of differences in continuity by economic background. For example, borrowing from physics and chemistry, the concept of metastable systems may be a fruitful framework for future research. In physics and chemistry, metastable systems are described as having multiple resting states and so these systems are stable at many different points [46]. Applied to the continuity of temporal selves, metastability may indicate a capacity for adaptability and openness to change in the self across time over a focus on a homogeneous self. While the current research found significant differences in the continuity of temporal self-perception by socioeconomic status, it is possible that students from different socioeconomic groups also vary in their perception of temporal selves as metastable. For example, college students from lower socioeconomic backgrounds may be more likely to perceive the relationship between their temporal selves as more adaptive and accommodating to change. Perceiving temporal selves as metastable— rather than simply continuous—may be predictive of positive long-term outcomes, particularly for students from a financially disadvantaged background.

Finally, we explored if both perceptions of the past self and perceptions of the future self were crucial predictors of psychological outcomes regardless of a student’s economic background. Our findings for depression symptoms and life satisfaction suggested an additive effect of past and future self-perceptions. Perceiving the past and future self as similar, vivid, and positive were both important predictors of fewer depression symptoms and greater satisfaction with life. This finding held regardless of a student’s socioeconomic status.

Contrastingly, in terms of meaning in life, we found a significant difference in socioeconomic status. Compared to financially disadvantaged students, for higher SES students, higher continuity in both temporal directions predicted greater meaning in life. For students from low socioeconomic backgrounds, temporal self-perceptions, regardless of temporal direction, were not predictive of greater meaning in life. Middle-class students seemed to fall between these two extremes. Similar to lower SES students, perceptions of the past self did not significantly predict meaning in life for middle-class students. However, unlike those from lower SES backgrounds, perceptions of the future self were important for understanding meaning in life for middle SES students. These findings were unexpected and may suggest that students from different economic backgrounds may differ in how they assess their meaning in life. Our results suggest that, for high SES students, perceptions of their temporal selves play an important role in their assessments of the presence of meaning in their lives. However, for students from lower SES backgrounds, this was not the case. These students may be considering other factors, and downplaying the role of the self when assessing their meaning in life. For middle SES students, they appear to be considering only the perception of the relationship between their present and future selves, and downplaying the role of the past, in their meaning in life. A measure of meaning in life was only available in one of our samples and this finding was exploratory. Future research should seek to replicate this unexpected finding and further explore the potential reasons for this difference by socioeconomic status.

## Figures and Tables

**Table 1:** Descriptive statistics.

		Full Sample			Low SES			Middle SES			High SES		
Variable	Sample	*M*	*SD*	*n*	*M*	*SD*	*n*	*M*	*SD*	*n*	*M*	*SD*	*n*
Perception of Future Self	1	4.93	1.00	391	4.85	0.98	80	4.94	0.98	152	4.96	1.05	154
	2	4.93	0.96	257	4.94	0.75	41	4.87	0.90	118	4.98	1.11	94
Future Similarity/Connect	1	4.21	1.36	391	3.82	1.30	80	4.26	1.37	152	4.32	1.37	154
	2	4.40	1.28	257	4.60	1.16	41	4.41	1.18	118	4.31	1.47	94
Future Positivity	1	5.89	0.97	391	5.87	0.97	80	5.86	1.01	152	5.95	0.93	154
	2	5.64	1.00	257	5.56	0.91	41	5.54	0.97	118	5.80	1.06	94
Future Vividness	1	4.70	1.59	391	4.87	1.42	80	4.70	1.52	152	4.62	1.74	154
	2	4.74	1.42	257	4.67	1.25	41	4.66	1.33	118	4.83	1.60	94
Perception of Past Self	1	3.97	1.00	391	3.72	0.92	80	4.02	0.97	151	4.06	1.04	154
	2	4.09	0.97	257	3.95	1.01	41	4.06	0.89	118	4.18	1.06	94
Past Similarity/Connect	1	3.03	1.35	391	2.66	1.16	80	3.03	1.25	151	3.22	1.48	154
	2	3.21	1.26	257	3.00	1.19	41	3.25	1.16	118	3.23	1.41	94
Past Positivity	1	3.88	1.52	391	3.46	1.42	80	3.86	1.54	151	4.12	1.51	154
	2	4.11	1.40	257	3.89	1.35	41	4.15	1.27	118	4.13	1.59	94
Past Vividness	1	5.01	1.47	391	5.03	1.43	80	5.16	1.46	151	4.84	1.49	154
	2	4.95	l.35	257	4.96	1.51	41	4.77	1.32	118	5.18	1.32	94
Depression	1	18.04	10.64	389	21.29	11.44	80	18.22	10.61	151	16.33	9.90	152
	2	18.48	10.78	256	20.34	10.90	41	17.77	10.07	118	18.82	11.66	93
Satisfaction with Life	1	--	--	--	--	--	--	--	--	--	--	--	--
	2	4.65	1.30	256	4.31	1.23	41	4.57	1.16	118	4.88	1.42	93
Meaning in Life	1	--	--	--	--	--	--	--	--	--	--	--	--
	2	4.77	1.23	256	4.52	1.24	41	4.80	1.16	118	4.83	1.30	93

Note. *M* = Mean. *SD* = Standard deviation. *n* = Sample size.

**Table 3:** Perceptions of the past and the future self predicting psychological outcomes.

Perceptions of the Past and the Future Self Predicting Depression										
	*df*	*F*	*p*	*R* ^2^	Effect	*b*	*SE*	*p*	95% CI	
									LL	UL
Sample 1	2, 388	24.94	<0.001	0.11						
					Intercept	2.91	0.15	<0.001	2.62	3.21
					Perception of the Past	−0.06	0.03	0.016	−0.11	−0.01
					Perception of the Future	−0.16	0.03	<0.001	−0.21	−0.10
Sample 2	2, 253	24.58	<0.001	0.16						
					Intercept	3.19	0.18	<0.001	2.83	3.56
					Perception of the Past	−0.13	0.03	<0.001	−0.19	−0.06
					Perception of the Future	−0.15	0.03	<0.001	−0.22	−0.09
Perceptions of the Past and the Future Self Predicting Meaning in Life										
	*df*	*F*	*p*	*R* ^2^	Effect	*b*	*SE*	*p*	95% CI	
									LL	UL
Sample 2	2, 253	30.73	<0.001	0.20						
					Intercept	1.66	0.41	<0.001	0.85	2.47
					Perception of the Past	0.16	0.08	0.029	0.02	0.31
					Perception of the Future	0.50	0.08	<0.001	0.35	0.64
Perceptions of the Past and the Future Self Predicting Satisfaction with Life										
	*df*	*F*	*p*	*R* ^2^	Effect	*b*	*SE*	*p*	95% CI	
									LL	UL
Sample 2	2, 253	32.98	<0.001	0.21						
					Intercept	1.24	0.43	0.004	0.39	2.09
					Perception of the Past	0.22	0.08	0.005	0.07	0.38
					Perception of the Future	0.51	0.08	<0.001	0.35	0.66

Note. Sample 1: *N* = 391; Sample 2: *N* = 257; LL = Lower Confidence Interval; UL = Upper Confidence Interval.

**Table 4:** Perception of the past self, socioeconomic status, and their interactions. Predicting perception of the future self.

	*df*	*F*	*p*	*R* ^2^	Effect	*b*	*SE*	*p*	95% CI	
									LL	UL
Sample 1	5, 380	5.56	<0.001	0.07						
					Intercept	4.89	0.11	<0.001	4.67	5.11
					PPS	0.14	0.12	0.256	−0.10	0.37
					SES: Low vs. Middle	0.05	0.14	0.733	−0.23	0.32
					SES: Low vs. High	0.04	0.14	0.769	−0.23	0.32
					PPS*Low vs. Middle	−0.06	0.15	0.658	−0.35	0.22
					PPS*Low vs. High	0.24	0.14	0.089	−0.04	0.52
	Increase in Variance Explained by Including Overall Interaction									
	*df*	*F*	*p*	Δ*R*^2^						
	2, 380	4.07	0.018	0.02						
	*df*	*F*	*p*	*R* ^2^	Effect	*b*	*SE*	*p*	95% CI	
									LL	UL
Sample 2	5, 247	6.54	<0.001	0.12						
					Intercept	4.95	0.14	<0.001	4.67	5.24
					PPS	0.07	0.14	0.616	−0.21	0.35
					SES: Low vs. Middle	−0.08	0.17	0.637	−0.41	0.25
					SES: Low vs. High	−0.02	0.17	0.923	−0.36	0.32
					PPS*Low vs. Middle	0.12	0.17	0.496	−0.22	0.45
					PPS*Low vs. High	0.40	0.17	0.019	0.07	0.73
	Increase in Variance Explained by Including Overall Interaction									
	*df*	*F*	*p*	Δ*R*^2^						
	2, 247	3.77	0.024	0.03						

Note. SES = Socioeconomic Status; PPS = Perception of the Past Self; PPS*Low vs. Middle = Interaction between perception of the past self and SES (low versus middle SES) predicting perception of the future self. PPS*Low vs. High = Interaction between perception of the past self and SES (low versus high SES) predicting perception of the future self. Δ*R*^2^ = Change in *R*^2^ by including the overall interaction in the regression equation.

**Table 5:** Simple slope analyses for perception of the past self predicting perception of the future self by socioeconomic status.

	SES Group	*b*	*SE*	*p*	95% CI	
					LL	UL
Sample 1						
	High SES	0.38	0.08	<0.001	0.23	0.53
	Middle SES	0.07	0.08	0.381	−0.09	0.23
	Low SES	0.14	0.12	0.256	−0.10	0.37
Sample 2						
	High SES	0.47	0.09	<0.001	0.29	0.64
	Middle SES	0.19	0.09	0.048	0.00	0.37
	Low SES	0.07	0.14	0.616	−0.21	0.35

Note. SES = Socioeconomic Status.

**Table 6:** Past self-similarity/connectedness, SES, and their interaction predicting future self-similarity/connectedness.

	*df*	*F*	*p*	*R* ^2^	Effect	*b*	*SE*	*p*	95% CI	
									LL	UL
Sample 1	5, 380	10.29	<0.001	0.12						
					Intercept	3.90	0.15	<0.001	3.60	4.19
					Past Self-Sim/Conn.	0.21	0.12	0.094	−0.04	0.45
					SES: Low vs. Middle	0.36	0.18	0.049	0.00	0.73
					SES: Low vs. High	0.34	0.18	0.063	−0.02	0.70
					PSC*Low vs. Middle	−0.07	0.15	0.644	−0.37	0.23
					PSC*Low vs. High	0.22	0.14	0.124	−0.06	0.50
	Increase in Variance Explained by Including Overall Interaction									
	*df*	*F*	*p*	Δ*R*^2^						
	2, 380	3.78	0.024	0.02						
	*df*	*F*	*p*	*R* ^2^	Effect	*b*	*SE*	*p*	95% CI	
									LL	UL
Sample 2	5, 247	9.35	<0.001	0.40						
					Intercept	4.66	0.19	<0.001	4.29	5.03
					Past Self-Sim/Conn.	0.30	0.16	0.063	−0.02	0.61
					SES: Low vs. Middle	−0.26	0.22	0.233	−0.69	0.17
					SES: Low vs. High	−0.36	0.23	0.113	−0.81	0.09
					PSC*Low vs. Middle	−0.09	0.19	0.639	−0.45	0.28
					PSC*Low vs. High	0.24	0.18	0.196	−0.12	0.59
	Increase in Variance Explained by Including Overall Interaction									
	*df*	*F*	*p*	Δ*R*^2^						
	2, 247	3.24	0.041	0.02						

Note. SES = Socioeconomic Status; PSC = Past Self-Similarity/Connectedness; PSC*Low vs. Middle = Interaction between past self-similarity/connectedness and SES (low versus middle-class status) predicting future self-similarity/connectedness. PSC*Low vs. High = Interaction between past self-similarity/connectedness and SES (low versus high SES) predicting future self-similarity/connectedness.

**Table 7:** Simple slope analyses for past self-similarity/connectedness predicting future self-similarity/connectedness by SES.

	SES Group	*b*	*SE*	*p*	95% CI	
					LL	UL
Sample 1						
	High SES	0.43	0.43	<0.001	0.29	0.57
	Middle SES	0.14	0.08	0.097	0.03	0.30
	Low SES	0.21	0.12	0.094	−0.04	0.45
Sample 2						
	High SES	0.53	0.09	<0.001	0.36	0.70
	Middle SES	0.21	0.10	0.029	0.02	0.40
	Low SES	0.30	0.16	0.063	−0.02	0.61

Note. SES = Socioeconomic Status.

**Table 8:** Past self-vividness, SES, and their interaction predicting future vividness.

	*df*	*F*	*p*	*R* ^2^	Effect	*b*	*SE*	*p*	95% CI	
									LL	UL
Sample 1	5, 380	2.64	0.023	0.03						
					Intercept	4.87	0.18	<0.001	4.52	5.21
					Past Self-Vividness	0.12	0.12	0.348	−0.13	0.36
					SES: Low vs. Middle	−0.17	0.22	0.434	−0.60	0.26
					SES: Low vs. High	−0.20	0.22	0.348	−0.63	0.22
					PSV*Low vs. Middle	−0.06	0.15	0.697	−0.36	0.24
					PSV*Low vs. High	0.16	0.15	0.284	−0.13	0.46
	Increase in Variance Explained by Including Overall Interaction									
	*df*	*F*	*p*	Δ*R*^2^						
	2, 380	1.69	0.185	0.01						
	*df*	*F*	*p*	*R* ^2^	Effect	*b*	*SE*	*p*	95% CI	
									LL	UL
Sample 2	5, 247	1.93	0.008	0.06						
					Intercept	4.67	0.22	<0.001	4.24	5.10
					Past Self-Vividness	0.04	0.15	0.763	−0.24	0.33
					SES: Low vs. Middle	0.03	0.25	0.897	−0.47	0.53
					SES: Low vs. High	0.09	0.26	0.736	−0.43	0.60
					PSV*Low vs. Middle	0.21	0.18	0.237	−0.14	0.55
					PSV*Low vs. High	0.27	0.18	0.140	−0.09	0.63
	Increase in Variance Explained by Including Overall Interaction									
	*df*	*F*	*p*	Δ*R*^2^						
	2, 247	1.13	0.324	0.01						

Note. SES = Socioeconomic Status; PSV = Past Self-Vividness; PV*Low vs. Middle = Interaction between Past Self-Vividness and SES (low versus middle SES) predicting Future Self-Vividness. PV*Low vs. High = Interaction between Past Vividness and SES (low versus high class status) predicting Future Vividness.

**Table 9:** Past self-positivity, SES, and their interaction predicting future self-positivity.

	*df*	*F*	*p*	*R* ^2^	Effect	*b*	*SE*	*p*	95% CI	
									LL	UL
Sample 1	5, 380	1.24	0.290	0.02						
					Intercept	5.90	0.11	<0.001	5.68	6.12
					Past Self-Positivity	0.06	0.08	0.399	−0.09	0.22
					SES: Low vs. Middle	−0.05	0.14	0.731	−0.32	0.22
					SES: Low vs. High	0.03	0.14	0.856	−0.25	0.30
					PSP*Low vs. Middle	−0.07	0.09	0.458	−0.25	0.11
					PSP*Low vs. High	0.05	0.09	0.616	−0.14	0.23
	Increase in Variance Explained by Including Overall Interaction									
	*df*	*F*	*p*	Δ*R*^2^						
	2, 380	1.26	0.285	0.01						
	*df*	*F*	*p*	*R* ^2^	Effect	*b*	*SE*	*p*	95% CI	
									LL	UL
Sample 2	5, 247	3.11	0.010	0.06						
					Intercept	5.54	0.15	<0.001	5.24	5.85
					Past Self-Positivity	−0.09	0.11	0.458	−0.31	0.14
					SES: Low vs. Middle	0.00	0.18	0.999	−0.35	0.35
					SES: Low vs. High	0.24	0.18	0.180	−0.12	0.61
					PSP*Low vs. Middle	0.07	0.13	0.588	−0.19	0.34
					PSP*Low vs. High	0.30	0.13	0.024	0.04	0.56
	Increase in Variance Explained by Including Overall Interaction									
	*df*	*F*	*p*	Δ*R*^2^						
	2, 247	4.04	0.019	0.03						

Note. SES = Socioeconomic Status; PSP = Past Self-Positivity; PSP*Low vs. Middle = Interaction between Past Self-Positivity and SES (low versus middle SES) predicting Future Self-Positivity. PSP*Low vs. High = Interaction between Past Self-Positivity and SES (low versus high-class status) predicting Future Self-Positivity.

**Table 10:** Simple slope analyses for past self-positivity predicting future self-positivity by SES.

	SES Group	*b*	*SE*	*p*	95% CI	
					LL	UL
Sample 1						
	High SES	0.11	0.05	0.032	0.01	0.21
	Middle SES	−0.004	0.05	0.941	−0.10	0.10
	Low SES	0.06	0.07	0.399	−0.09	0.22
Sample 2						
	High SES	0.21	0.06	0.001	0.09	0.34
	Middle SES	−0.01	0.07	0.868	−0.15	0.13
	Low SES	−0.09	0.11	0.458	−0.31	0.14

Note. SES = Socioeconomic Status.

**Table 11:** Temporal self-perceptions, SES, and interaction predicting meaning in life.

Perception of the Past Self, SES, and their Interactions Predicting Meaning in Life										
	*df*	*F*	*p*	*R* ^2^	Effect	*b*	*SE*	*p*	95% CI	
									LL	UL
Sample 2	5, 246	4.82	<0.001	0.09						
					Intercept	4.53	0.19	<0.001	4.16	4.90
					PPS	0.07	0.18	0.702	−0.29	0.43
					SES: Low vs. Middle	0.27	0.22	0.207	−0.15	0.70
					SES: Low vs. High	0.25	0.22	0.257	−0.19	0.69
					PPS*Low vs. Middle	0.11	0.22	0.618	−0.33	0.55
					PPS*Low vs. High	0.45	0.22	0.042	0.02	0.87
	Increase in Variance Explained by Including Overall Interaction									
	*df*	*F*	*p*	Δ*R*^2^						
	2, 246	2.96	0.054	0.02						
Perception of the Future Self, SES, and their Interaction Predicting Meaning in Life										
	*df*	*F*	*p*	*R* ^2^	Effect	*b*	*SE*	*p*	95% CI	
									LL	UL
Sample 2	5, 246	12.26	<0.001	0.20						
					Intercept	4.52	0.17	<0.001	4.18	4.86
					PFS	0.03	0.23	0.911	−0.44	0.49
					SES: Low vs. Middle	0.31	0.20	0.130	−0.09	0.70
					SES: Low vs. High	0.27	0.21	0.192	−0.14	0.68
					PFS*Low vs. Middle	0.49	0.26	0.059	−0.02	1.01
					PFS*Low vs. High	0.61	0.26	0.018	0.11	1.12
	Increase in Variance Explained by Including Overall Interaction									
	*df*	*F*	*p*	Δ*R*^2^						
	2, 246	2.86	0.059	0.02						

Note. SES = Socioeconomic Status; PPS = Perception of the Past Self; PPS*Low vs. Middle = Interaction between Perception of the Past Self and SES (low versus middle-class status) predicting meaning in life. PPS*Low vs. High = Interaction between perception of the past self and SES (low versus high) predicting meaning in life. PFS = Perception of the Future Self; PFS*Low vs. Middle = Interaction between Perception of the Future Self and SES (low versus) predicting meaning in life. PFS*Low vs. High = Interaction between perception of the future self and SES (low versus high-class status) predicting meaning in life.

**Table 12:** Simple slope analyses for temporal self-perceptions predicting meaning in life by SES.

	SES Group	*b*	*SE*	*p*	95% CI	
					LL	UL
Perception of Past Self Predicting Meaning in Life						
Sample 2						
	High SES	0.52	0.12	<0.001	0.29	0.75
	Middle SES	0.18	0.12	0.142	−0.06	0.42
	Low SES	0.07	0.18	0.702	−0.29	0.43
Perception of Future Self Predicting Meaning in Life						
Sample 2						
	High SES	0.64	0.10	<0.001	0.43	0.84
	Middle SES	0.52	0.11	<0.001	0.30	0.74
	Low SES	0.03	0.23	0.911	−0.44	0.49

Note. SES = Socioeconomic Status.

## Data Availability

Deidentified data sets are available to download at https://osf.io/d5w8n/?view_only=da8f7c52ac6f429ca5509c3e5cdb0ffc (accessed on 7 July 2024).

## Supplementary Materials

The following supporting information can be downloaded at: https://www.mdpi.com/article/10.3390/bs14100858/s1, Table S1: Temporal Self Perceptions, Socioeconomic Status, and their Interactions Predicting Depression; Table S2: Temporal Self Perceptions, Socioeconomic Status, and their Interaction Predicting Satisfaction with Life; SES Crosstabulaiton tables.
