# Supplementary material for: Perceptions of Temporal Selves: Continuity, Psychological Outcomes, and the Significance of a Disadvantaged Background
Source: Behav Sci (Basel). 2024 Sep 24;14(10):858. doi: 10.3390/bs14100858 (PMC11505364; doi:10.3390/bs14100858)
Supplement: Supplementary file 1 [file behavsci-14-00858-s001.zip › behavsci-3121228-supplementary.pdf]

Perceptions of Temporal Selves: Continuity, Psychological Outcomes, and the Significance of a Disadvantaged Background

Supplemental Materials

Table S1

Temporal Self Perceptions, Socioeconomic Status, and their Interactions Predicting Depression

Perception of the Past Self, Socioeconomic Status, and their Interactions Predicting Depression									95% CI	
	df	*F*	*p*	R^2	Effect	*b*	*SE*	*p*	LL	UL
Sample 1	5, 378	4.84	<.001	0.06						
					Intercept	20.75	1.20	<.001	18.38	23.12
					PPS	-1.65	1.27	.196	-4.15	0.86
					SES: Low v Middle	-2.43	1.47	.099	-5.32	0.46
					SES: Low v High	-4.26	1.47	.004	-7.15	-1.36
					PPS*Low v Middle	-0.55	1.54	.720	-3.59	2.48
					PPS*Low v High	-0.85	1.51	.955	-3.05	2.88
Sample 2	5, 246	5.81	<.001	0.11						
					Intercept	19.78	1.63	<.001	16.57	22.98
					PPS	-4.34	1.61	.008	-7.51	-1.17
					SES: Low v Middle	-2.07	1.88	.274	-5.78	1.64
					SES: Low v High	-0.63	1.95	.746	-4.47	3.21
					PPS*Low v Middle	1.69	1.93	.383	-2.12	5.50
					PPS*Low v High	0.59	1.90	.757	-3.16	4.34

Perception of the Future Self, Socioeconomic Status, and their Interactions Predicting Depression									95% CI	
	df	*F*	*p*	R^2	Effect	*b*	*SE*	*p*	LL	UL
Sample 1	5, 378	10.46	<.001	0.12						
					Intercept	20.93	1.12	<.001	18.72	23.14
					PFS	-3.34	1.16	.004	-5.62	-1.06
					SES: Low v Middle	-2.66	1.39	.056	-5.39	0.06
					SES: Low v High	-4.52	1.39	.001	-7.24	-1.79
					PFS*Low v Middle	-0.19	1.43	.892	-3.00	2.61
					PFS*Low v High	0.51	1.39	.716	-2.23	3.25
Sample 2	5, 246	6.86	<.001	0.12						
					Intercept	20.42	1.60	<.001	17.28	23.57
					PFS	-3.76	2.16	.084	-8.02	0.50
					SES: Low v Middle	-2.83	1.86	.128	-6.49	0.82
					SES: Low v High	-1.37	1.92	.476	-5.15	2.41
					PFS*Low v Middle	0.30	2.40	.899	-4.43	5.04
					PFS*Low v High	-0.35	2.37	.884	-5.00	4.31

Note. SES = Socioeconomic Status; PPS = Perception of the Past Self; PPS*Low v Middle = Interaction between Perception of the Past Self and SES (low versus middle class status) predicting depression. PPS*Low v High = Interaction between perception of the past self and SES (low versus high class status) predicting depression. PFS = Perception of the Future Self; PFS*Low v Middle = Interaction between Perception of the Future Self and SES (low versus middle class status) predicting depression. PFS*Low v High = Interaction between perception of the future self and SES (low versus high class status) predicting depression.

Table S2

Temporal Self Perceptions, Socioeconomic Status, and their Interaction Predicting Satisfaction with Life

Perception of the Past Self, Socioeconomic Status, and their Interactions Predicting Satisfaction with Life									95% CI	
	df	*F*	*p*	R^2	Effect	*b*	*SE*	*p*	LL	UL
Sample 2	5, 246	5.64	<.001	0.10						
					Intercept	4.35	0.19	<.001	3.97	4.73
					PPS	0.28	0.19	.143	-0.10	0.66
					SES: Low v Middle	0.23	0.22	.309	-0.21	0.67
					SES: Low v High	0.49	0.23	.035	0.04	0.95
					PPS*Low v Middle	0.00	0.23	.990	-0.45	0.46
					PPS*Low v High	0.17	0.23	.445	-0.27	0.62

Perception of the Future Self, Socioeconomic Status, and their Interactions Predicting Satisfaction with Life									95% CI	
	df	*F*	*p*	R^2	Effect	*b*	*SE*	*p*	LL	UL
Sample 2	5, 246	12.51	<.001	0.20						
					Intercept	4.31	0.18	<.001	3.95	4.66
					PFS	0.32	0.25	.189	-0.16	0.81
					SES: Low v Middle	0.29	0.21	.168	-0.12	0.71
					SES: Low v High	0.54	0.22	.014	0.11	0.97
					PFS*Low v Middle	0.15	0.27	.579	-0.39	0.69
					PFS*Low v High	0.34	0.27	.209	-0.19	0.87

Note. SES = Socioeconomic Status; PPS = Perception of the Past Self; PPS*Low v Middle = Interaction between Perception of the Past Self and SES (low versus middle class status) predicting satisfaction with life. PPS*Low v High = Interaction between perception of the past self and SES (low versus high class status) predicting satisfaction with life. PFS = Perception of the Future Self; PFS*Low v Middle = Interaction between Perception of the Future Self and SES (low versus middle class status) predicting satisfaction with life. PFS*Low v High = Interaction between perception of the future self and SES (low versus high class status) predicting satisfaction with life.

Socioeconomic Status by Mother's Educational Attainment: Sample 1

SES	Less than High School	High school diploma (or GED)	Some college or a 2-year college degree (A.A.)	4-year college degree (B.A., B.S.)	Master's degree (M.A., M.S.)	Graduate or professional degree (J.D., Ph.D., M.D.)
Low	16	23	25	10	5	1
	53.30%	33.80%	31.60%	7.90%	8.20%	5.90%
Middle	13	29	29	50	28	1
	43.30%	42.60%	36.70%	39.70%	45.90%	5.90%
High	1	16	25	66	28	15
	3.30%	23.50%	31.60%	52.40%	45.90%	88.20%

Socioeconomic Status by Father's Educational Attainment: Sample 1

SES	Less than High School	High school diploma (or GED)	Some college or a 2-year college degree (A.A.)	4-year college degree (B.A., B.S.)	Master's degree (M.A., M.S.)	Graduate or professional degree (J.D., Ph.D., M.D.)
Low	22	27	9	11	4	3
	56.40%	39.70%	16.10%	9.20%	6.50%	8.30%
Middle	16	27	25	47	24	11
	41.00%	39.70%	44.60%	39.50%	38.70%	30.60%
High	1	14	22	61	34	22
	2.60%	20.60%	39.30%	51.30%	54.80%	61.10%

Socioeconomic Status by Mother's Educational Attainment: Sample 2

SES	Less than High School	High school diploma (or GED)	Some college or a 2-year college degree (A.A.)	4-year college degree (B.A., B.S.)	Master's degree (M.A., M.S.)	Graduate or professional degree (J.D., Ph.D., M.D.)
Low	7	18	8	5	1	2
	36.80%	32.10%	15.40%	6.70%	2.60%	16.70%
Mid	11	26	21	39	18	3
	57.90%	46.40%	40.40%	52.00%	46.20%	25.00%
High	1	12	23	31	20	7
	5.30%	21.40%	44.20%	41.30%	51.30%	58.30%

Socioeconomic Status by Father's Educational Attainment: Sample 2

SES	Less than High School	High school diploma (or GED)	Some college or a 2-year college degree (A.A.)	4-year college degree (B.A., B.S.)	Master's degree (M.A., M.S.)	Graduate or professional degree (J.D., Ph.D., M.D.)
Low	10	11	10	5	2	1
	58.80%	23.40%	22.20%	6.40%	4.40%	5.60%
Mid	5	25	20	39	24	4
	29.40%	53.20%	44.40%	50.00%	53.30%	22.20%
High	2	11	15	34	19	13
	11.80%	23.40%	33.30%	43.60%	42.20%	72.20%

The crosstabulation results were significant in both samples for both mother's and father's education (SES and Mother's Education: Sample 1: χ^2 (10, N = 381) = 78.932, p <.001; Sample 2: χ^2 (10, N = 253) = 38.630, p <.001; SES and Father's Education: Sample 1: χ^2 (10, N = 380) = 86.490, p <.001; Sample 2: χ^2 (10, N = 250) = 49.395, p <.001).
